# Unstable Lumbar Vertebral Body Fracture During Total Hip Arthroplasty Using the Anterolateral Spine Approach in Diffuse Idiopathic Skeletal Hyperostosis: A Case Report and Literature Review

**DOI:** 10.1016/j.artd.2025.101874

**Published:** 2025-10-04

**Authors:** Shinichi Ueki, Takeshi Shoji, Naosuke Kamei, Hiroki Kaneta, Hiroyuki Morita, Yosuke Kozuma, Naoto Nakayama, Nobuo Adachi

**Affiliations:** a Department of Orthopaedical Surgery, Graduate School of Biomedical Sciences, Hiroshima University, Minami, Hiroshima, Japan; b Faculty of Medicine, Division of Orthopaedic Surgery, Department of Medicine of Sensory and Motor Organs, University of Miyazaki, Kiyotake, Miyazaki, Japan

**Keywords:** Total hip arthroplasty, Anterolateral supine approach, Complication, Diffuse idiopathic skeletal hyperostosis, Lumbar vertebral body fracture

## Abstract

This case report describes a 72-year-old man with diffuse idiopathic skeletal hyperostosis who sustained a rare unstable lumbar vertebral body fracture during total hip arthroplasty (THA) using the anterolateral supine approach. Despite a successful THA, the patient developed severe postoperative back pain, leading to the diagnosis of a lumbar vertebral fracture. Prompt posterior spinal fusion effectively relieved the pain. This report emphasizes recognizing high-risk patients, such as those with diffuse idiopathic skeletal hyperostosis complicated by obesity or kyphosis, before THA, and underscores the need for careful perioperative management and intraoperative positioning to reduce stress on the lumbar spine.

## Introduction

Diffuse idiopathic skeletal hyperostosis (DISH) is a chronic disease characterized by calcification and ossification of the spinal ligaments and peripheral entheses [1,2]. In affected patients, vertebral fractures commonly result from low-energy trauma, such as falls from standing or sitting positions [3]. However, reports of vertebral fractures caused by intraoperative body positioning in individuals with ankylosing spinal disorders (ASDs) are limited. This case report describes a patient with DISH who sustained an unstable lumbar vertebral body fracture during total hip arthroplasty (THA) performed via the anterolateral supine approach. To the best of our knowledge, only 6 cases of vertebral body fractures during THA have been reported, with this case being the first associated with the anterolateral approach. Similar fractures have been documented using other techniques, such as the direct anterior and Hardinge techniques [[4], [5], [6], [7], [8]]. Despite variations in approach, previous studies have implicated supine lumbar hyperextension as a potential contributing factor to THA-associated fractures. Nevertheless, the precise mechanism remains unclear, and specific risk factors and preventive strategies have not been fully established. This case report aims to describe a rare complication of unstable lumbar vertebral body fracture occurring during THA using the anterolateral supine approach in a patient with DISH and to provide perioperative countermeasures based on insights from the existing literature.

## Case history

**Figure 1:**
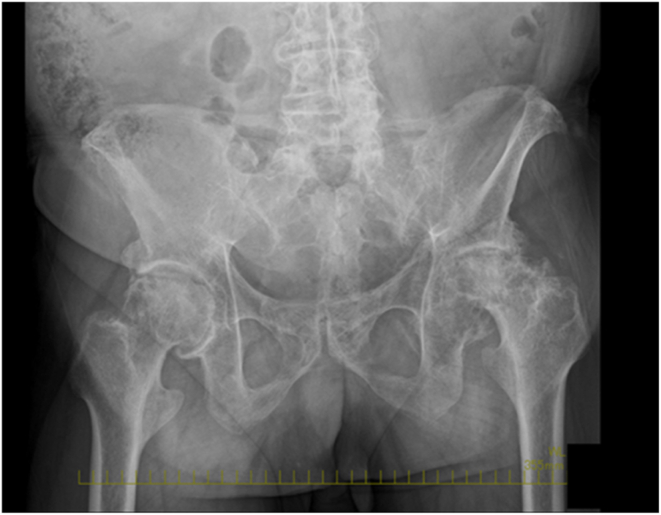
Preoperative pelvic radiograph.

**Figure 2:**
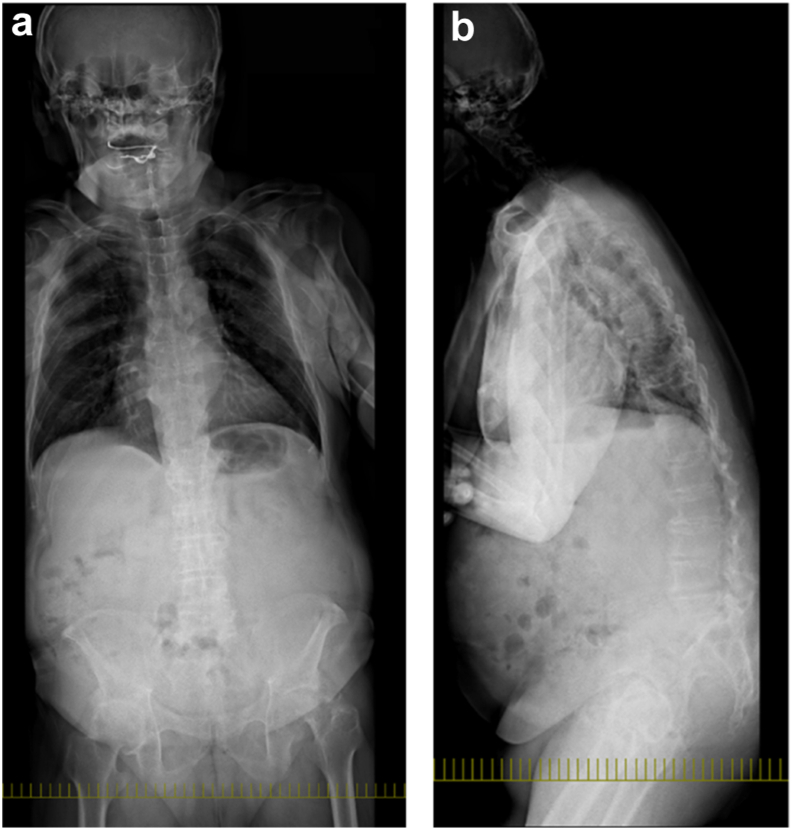
Preoperative full-spine radiograph. (a) Anteroposterior view and (b) Lateral view.

**Figure 3:**
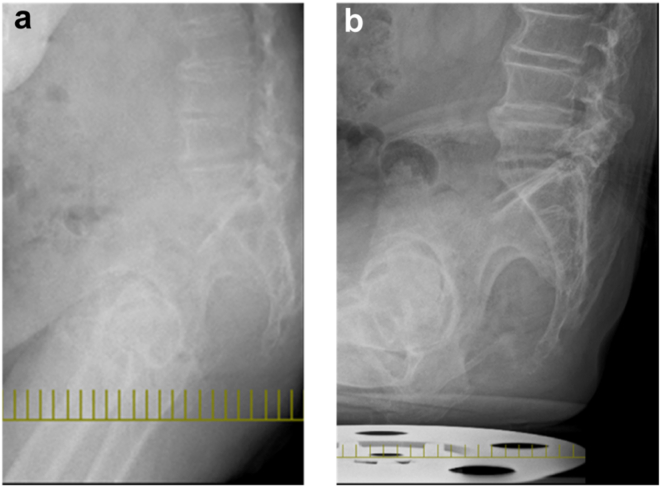
Lateral view of lumbar-pelvic radiograph. (a) SS measured at 11° in the standing position and (b) SS measured at 4° in the sitting position. Δ SS = 7°.

The patient gave written, informed consent to have this case report published. A 72-year-old man with left hip pain was referred to our hospital. His medical history included cervical spondylotic myelopathy, chronic atrial fibrillation, hypertension, diabetes mellitus (HbA1c: 7.5%), and obesity (body mass index: 32.1 kg/m^2^), classified as class I obesity according to WHO guidelines. A preoperative pelvic radiograph showed the severe narrowing of the left hip joint space, consistent with end-stage osteoarthritis (Fig. 1). At our institution, full-spine radiographs are routinely performed for all patients undergoing THA to assess spinal alignment and potential hip–spine relationships. A full-spine radiograph showed a pelvic tilt of 47°, pelvic incidence of 58°, sacral slope (SS) of 11°, lumbar lordosis of 9°, pelvic incidence–lumbar lordosis mismatch of 55°, sagittal vertical axis of 165 mm, marked pelvic retroversion, thoracolumbar kyphotic deformity, and anterior displacement of the load axis (Fig. 2). Additionally, a preoperative lateral lumbar-pelvic radiograph (Fig. 3) showed a 7° difference in SS (ΔSS) between the standing (a) and sitting (b) positions, indicating decreased lumbar-pelvic mobility consistent with a stiff spine [9]. The preoperative range of motion (right/left) was as follows: flexion 120°/80°, extension −25°/−45°, abduction 20°/0°, adduction 20°/10°, external rotation 45°/40°, internal rotation 0°/−20°, with markedly limited flexion and extension.

**Figure 4:**
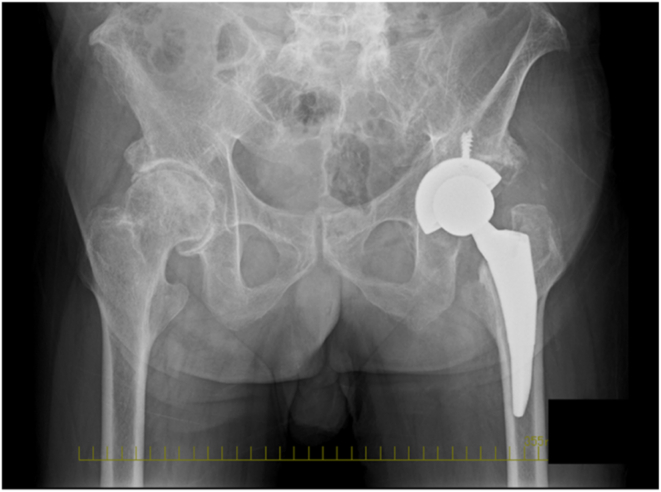
Postoperative pelvic radiograph.

The patient, diagnosed with end-stage osteoarthritis of the left hip, underwent left THA using the anterolateral supine approach under general anesthesia. Surgery was performed on a standard table, with a cushion approximately 10 cm in height placed under the sacrum to facilitate effective hip extension. Acetabular cup placement was guided by a computed tomography (CT)-based navigation system (Hip version 1.0; Stryker Navigation, Freiburg, Germany). Our institution routinely employs either a CT-based navigation system or a robotic-assist system for all THA procedures. The femoral stem was inserted with the surgical table extended by approximately 30°. Both the vertical and horizontal bands of the iliofemoral ligament were carefully detached from the femur to allow sufficient extension and external rotation. Implanted components included an uncemented porous-coated hemispherical cup, an uncemented tapered wedge stem, and a 36-mm delta-ceramic head. The operative time was 100 min. The surgery was completed without complications (Fig. 4).

**Figure 5:**
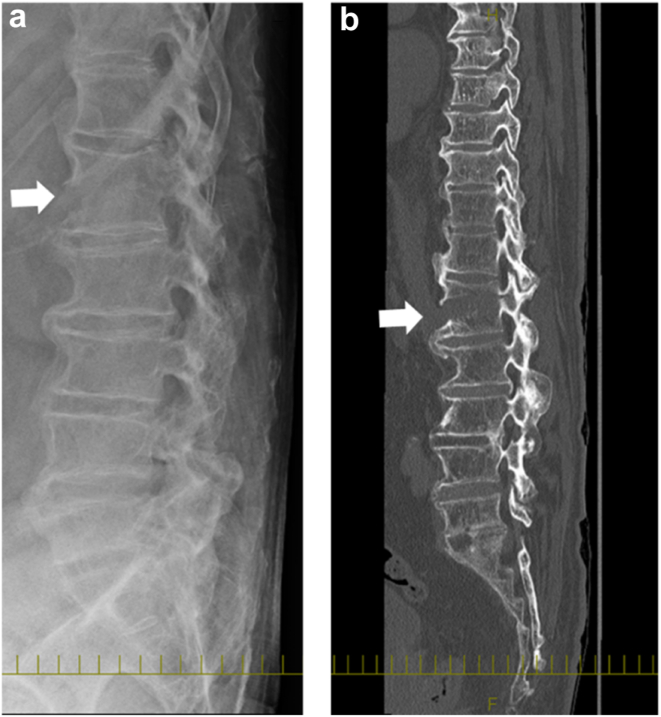
Postoperative spine radiograph and CT. (a) Lateral view of radiograph and (b) Lateral view of CT. White arrows indicate the fracture line extending to the posterior column.

**Figure 6:**
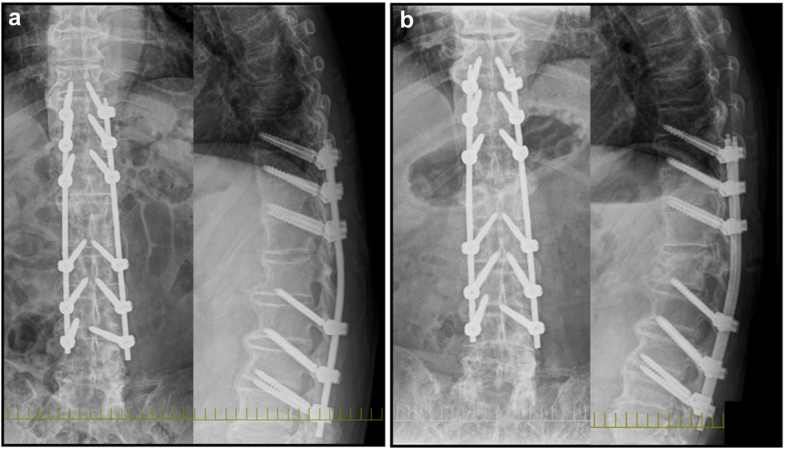
Postoperative spine radiograph after posterior spinal fusion. (a) Immediately after surgery and (b) One year after surgery.

Upon emergence from anesthesia, the patient was transferred to the inpatient ward after confirming intact ankle joint mobility and preserved sensation in the lower extremities. Shortly after arrival, he reported severe back pain, rated as 8 on the Visual Analog Scale. Although postoperative back pain is relatively common, its persistence and severity, coupled with the patient's inability to stand even after 3 days, warranted further investigation. A lumbar spine radiograph revealed a fracture of the first lumbar vertebra, which was confirmed by CT demonstrating a fracture line extending into the posterior column, along with evidence of DISH characterized by ossification of the thoracolumbar anterior longitudinal ligament (Fig. 5). After obtaining informed consent, the patient underwent posterior spinal fusion from Th10 to L4 on postoperative day 7. This intervention led to rapid relief of back pain and restoration of ambulation. At the 1-year follow-up, bone union was confirmed (Fig. 6).

## Discussion

This report describes a patient with DISH and marked obesity, kyphosis, and limited hip extension, who developed an unstable lumbar vertebral body fracture during THA using anterolateral supine approach, requiring posterior spinal fusion. To the best of our knowledge, only 6 cases of vertebral fractures during THA have been previously reported. Table 1 shows the clinical characteristics of those cases alongside the present case. Most reported cases occurred in patients with ASD, DISH, or ankylosing spondylitis (AS) [[4], [5], [6], [7]]. However, vertebral fractures have also been observed in patients without DISH or AS but with stiff spines, as indicated by a ΔSS of 10° [8]. In this case, the patient had a significantly stiff lumbar spine with ossification of the anterior longitudinal ligament and a ΔSS of 7° on preoperative radiographs. These findings suggest the patient was at high risk for lumbar extension-induced vertebral fracture, highlighting the need for greater intraoperative caution in similar cases.

**Table 1:** Patient characteristics in previous reports and the present case.

Author	Age (y)	Sex	Medical history	THA approach	Position	Fracture level	Kyphosis
Danish S F. [4]	59	Female	AS, obesity	Hardinge	Supine	Th11-12	+
	60	Male	AS, LCS, obesity	Hardinge	Supine	Th11	+
Königshausen M. [5]	57	Female	DISH, LCS, obesity	Lateral	Supine	Th11	Not described
Pitta M. [6]	68	Male	AS	Direct anterior	Supine	L4-5	+
Petis S. [7]	90	Male	DISH	Direct anterior	Supine	L1	+
Mosich GM [8].	86	Male	obesity	Direct anterior	Supine	Th12-L1	Not described
This case	72	Male	DISH, obesity	Anterolateral	Supine	L1	+

LCS, lumbar canal stenosis; Th, thoracic spine; L, lumbar spine.

The underlying causes of DISH remain largely unclear. However, excessive osteoblast proliferation, likely caused by a combination of genetic disposition and external factors, such as environmental influences, obesity, and metabolic conditions, such as diabetes mellitus, has been implicated in its pathogenesis [10]. As DISH is closely associated with metabolic dysregulation, dietary patterns, and aging population [11], the increasing number of elderly patients undergoing THA is predicted to contribute to a rise in complications, such as lumbar vertebral fractures. Furthermore, lumbar vertebral fractures in patients with ASD and severe kyphosis have been reported not only intraoperatively during THA but also postoperatively in the supine position, as well as during intramedullary nailing for femoral trochanteric fractures performed on traction tables [12,13]. This case report underscores the critical importance of prioritizing lateral positioning in patients with DISH whenever possible. In an increasingly aging society, surgical candidates often present for surgery with complex comorbidities and diverse risk profiles. Furthermore, hyperextension fractures in patients with DISH typically result from low-energy trauma [14], underscoring the need to avoid spinal hyperextension during perioperative and intraoperative procedures, regardless of the type of surgery. In this case, the patient's obesity posed an additional risk factor. A retrospective study involving 965 patients with thoracic spine hyperextension injuries found that all were obese, with an average body mass index of 32.7 kg/m^2^ [15]. This indicates that obesity itself is a major risk factor for thoracolumbar spine injury, even in the absence of ASD.

Generally, THA using anterolateral supine approach is associated with a lower rate of dislocation, shorter hospital stays, and faster early postoperative recovery [16,17]. However, achieving optimal femoral stem placement in THA using anterolateral supine approach requires extension of the lumbar spine and hip joint. The supine position poses a high risk of spinal fractures in patients with AS [18]. For patients with ASD and obesity, kyphosis, or severe hip extension limitations, obtaining informed consent regarding potential vertebral fracture risk is important. Surgeons should perform lumbar extension with great care during the procedure. In high-risk patients, it may be advisable to select a posterior approach in the lateral position utilizing hip flexion, adduction, and internal rotation to facilitate femoral stem placement. Moreover, once patients at risk (those with ASD, obesity, or kyphosis) are identified, meticulous attention must be paid to patient transfer, positioning, and intraoperative handling, particularly under general anesthesia, when protective neuromuscular feedback is diminished.

**Figure 7:**
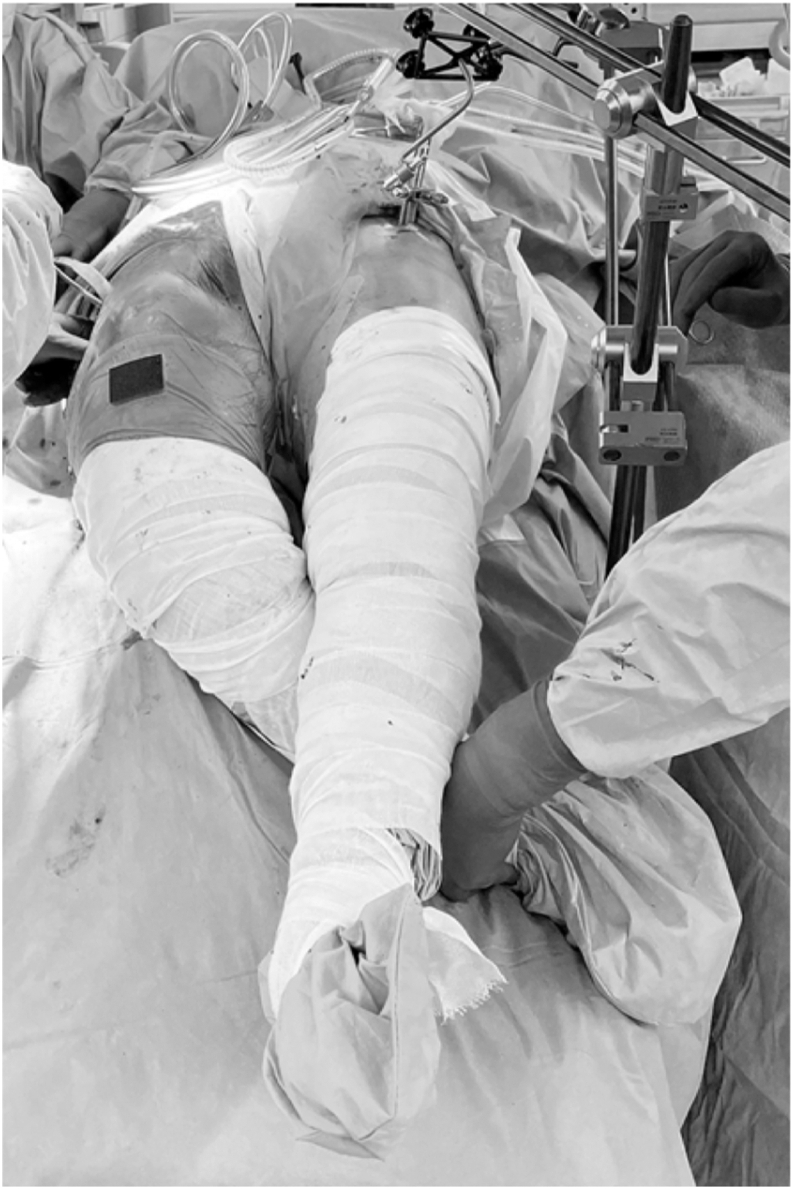
Modified position commonly referred to as the “figure-of-4” position.

Nevertheless, since the hip joint is disarticulated during intraoperative hip extension, the exact amount of force transmitted to the spine remains unclear, even when the patient is in the supine position. We hypothesized that extension of the nonoperative limb increases extensional stress on the lumbar spine. Currently, we have revised the positioning that previously resulted in hyperextension of both hips. We now sterilize the nonoperative limb and position the operative limb beneath it during femoral stem placement. This new configuration prevents hyperextension of the nonoperative hip and is commonly referred to as the “figure-of-4” position (Fig. 7).We recommend this position in cases involving individuals with a history of spinal disorders, those with a stiff spine and decreased ΔSS, and other high-risk individuals, such as those with pre-existing kyphosis, advanced age, or obesity, as supported by existing literature. Although no vertebral fractures have been observed thus far, the limited number of THA cases performed with this technique necessitates further investigation to establish its efficacy.

Our case represents the first report of an unstable vertebral fracture occurring in THA utilizing a CT-based navigation system for acetabular component placement. Navigation system is designed to enhance the accuracy of implant positioning and generally does not require additional exposure or manipulation beyond the standard anterolateral supine approach. The operative time in this case (100 minutes) was within the expected range for complex THA and was not prolonged due to the use of navigation itself. Therefore, we believe that navigation did not directly contribute to the occurrence of the vertebral body fracture. Nonetheless, it is imperative for surgeons utilizing computer-assisted procedures to acknowledge the potential for such complications in THA with navigation and to recognize the importance of preoperative spinal evaluation.

## Summary

This case report describes a rare complication of an unstable lumbar vertebral body fracture in a 72-year-old man with DISH, obesity, and kyphosis during THA using the anterolateral supine approach. This case highlights the risks associated with supine positioning and lumbar extension in patients with ankylosed spines and limited pelvic mobility. The authors emphasize that patients with DISH, obesity, or severe kyphosis are at increased risk for unstable lumbar vertebral fractures, and intraoperative positioning should be carefully managed to minimize spinal stress.

## Conflicts of interest

The authors declare there are no conflicts of interest.

For full disclosure statements refer to https://doi.org/10.1016/j.artd.2025.101874

## Informed patient consent

The author confirmed that written informed consent has been obtained from the patient involved. Therefore, he has given approval for this information to be published in this case report.

## CRediT authorship contribution statement

**Shinichi Ueki:** Writing – original draft, Data curation, Conceptualization. **Takeshi Shoji:** Writing – review & editing, Project administration. **Naosuke Kamei:** Writing – review & editing. **Hiroki Kaneta:** Writing – review & editing, Conceptualization. **Hiroyuki Morita:** Writing – review & editing. **Yosuke Kozuma:** Writing – review & editing. **Naoto Nakayama:** Writing – review & editing. **Nobuo Adachi:** Writing – review & editing.

## Appendix A Supplementary data

Conflict of Interest Statement for all authors
